# Biplanar EOS screening in children with hereditary multiple osteochondromas: a feasible screening method?

**DOI:** 10.3389/fped.2025.1625991

**Published:** 2026-01-12

**Authors:** Henrik Hedelin, Jenny Ahlin, Aina Danielsson, Helena Brisby, Kerstin Lagerstrand

**Affiliations:** 1 Department of Orthopaedics, Sahlgrenska University Hospital, Gothenburg, Sweden; 2 Institute of Clinical Sciences, Sahlgrenska Academy, University of Gothenburg, Gothenburg, Sweden; 3 Department of Radiology, Sahlgrenska University Hospital, Gothenburg, Sweden; 4 Department of Biomedical Engineering and Medical Physics, Sahlgrenska University Hospital, Gothenburg, Sweden

**Keywords:** biplanar x-rays, children, EOS, hereditary multiple exostoses, hereditary multiple osteochondromas

## Abstract

**Background:**

Children with Hereditary Multiple Ostechondromas (HMO) require regular screening to identify gradual dysplasia or osteochondromas that need surgery. EOS biplanar imaging enables interpretation of weight-bearing malalignment with low ionizing radiation exposure.

**Objective:**

To determine whether EOS can be used as a screening method for presence of osteochondromas and frontal plane malalignment in the lower extremities in HMO children.

**Methods:**

Presence of ostechondromas was determined in the lower extremities in ten children [age: 14.5(10–20) years, six males] using EOS. Also, frontal plane malalignment was registered. Further, patient reported experience measures (PREM) were collected through a questionnaire. EOS findings were compared with conventional whole-leg radiographs. The frontal plane malalignment was determined using standardized measurements for hip, knee and ankle joints. Reproducibility for both modalities was determined through repeated measurements by the same observer.

**Results:**

EOS systematically identified significantly more osteochondromas than conventional frontal radiographs in the lower limbs. Measurements of malalignment in the frontal plane showed only a small systematic bias with narrow limits of agreement between the modalities with mean difference −0.86 degrees (95% LOA: −6.36–4.63 degrees). Intra-observer agreement for both modalities was very good (CV_radiographs_ = 2%; CV_EOS_ = 1%;). The PREM findings showed that EOS was well-tolerated by the children.

**Conclusions:**

EOS biplanar imaging was well-tolerated and seemed to be a feasible alternative to conventional radiographs for screening of children with HMO, offering reduced radiation exposure and the potential to detect more osteochondromas in the lower limbs.

## Background

In children with hereditary multiple osteochondromas (HMO), benign skeletal osteochondromas typically grow during childhood and adolescence (1–3). While most osteochondromas remain completely benign, there is a lifetime risk of 2%–5% having at least one malignant transformation (4). Also, malignant transformations almost never occur during childhood and very rarely during the teenage period (4, 5).

Both children and adults with HMO require regular radiographic screening. Malignancy is the main indication for adults, whereas in children the primary goal is to avoid gradual dysplasia caused by the osteochondromas. In children, HMO commonly cause frontal plane malalignments in the ankle, knee and hip, either because of tethering or displacement due to the tumor mass (3, 6–8). Common malalignments include genu valgum and valgus deformity of the ankle. In the hip, coxa valga is frequent and may lead to poor acetabular coverage of the femoral head and secondary dysplasia or instability of the hip joint (1, 7). For both malalignments and local lesion problems, surgery can often correct or prevent the condition, but delayed diagnosis often requires more extensive surgery. The different indications for radiologic screening in adults and children are of essence when choosing both radiographic modality and frequency.

For children with HMO, there is no universally accepted screening protocol. Standing whole leg radiographs have the possibility to map weight-bearing malalignment of the hip, knee and ankle in the frontal plane and to identify bone lesions visible in the same plane. Both CT and MRI are capable of detecting osteochondromas in various planes. However, these examinations are typically performed in supine position and, as such, do not provide visualizations of weight-bearing position nor true malalignment and correct acetabular coverage of the femoral head (9). Further, CT scans entail substantial radiation exposure (10). Considering the main indications for monitoring HMO in children, a low-dose screening method for imaging of the entire body in a weight-bearing position would be optimal.

EOS biplanar imaging may provide such tool with a markedly low ionizing radiation exposure. EOS® imaging system is also known as a slot-scanning device or slit-beam digital radiography system. The x-ray technology allows acquisition of simultaneous AP and lateral images of the entire body in an erect position. The method has been used for numerous indications, including scoliosis and lower limb alignment (11–14). In children, EOS has been proven to reliably estimate both limb length, torsional parameters, and alignment in the frontal plane of the lower limbs (15, 16).

The aim of the present study was to determine whether EOS biplanar imaging is a feasible tool for bone lesion screening and identification of lower limb malalignment in children with HMO, using standard weight-bearing frontal radiographs and the child's own experience as references.

## Methods

### Study group

Fourteen children with a diagnosis of HMO who attended our clinic for routine follow-up between February 2023 and June 2023 were invited to participate. Children were defined by open physis in the long bones and the exclusion criteria were lack of consent or planned elective surgery during the study period.

As per routine at our department and according to national guidelines the HMO diagnosis had previously been made by the combination of family history and more than three typical osteochondromas. In our study group none of the patients had undergone genetic testing.

### Examination

In conjunction with the scheduled follow-up appointment with a senior pediatric orthopedic surgeon, all included children underwent a conventional weight-bearing whole leg radiography as part of the standard follow-up protocol. The conventional weight-bearing whole leg radiography was performed on a fully digital system (Canon Adora DRi and Arcoma Precision i5). According to the patient's weight and stature, different preset tube voltages were chosen by the radiographer (small, 75–85 kV; medium, 77–90 kV; large, 77–96 kV). To reduce the radiation exposure, the standard follow-up protocol did not include lateral views.

**Figure 1:**
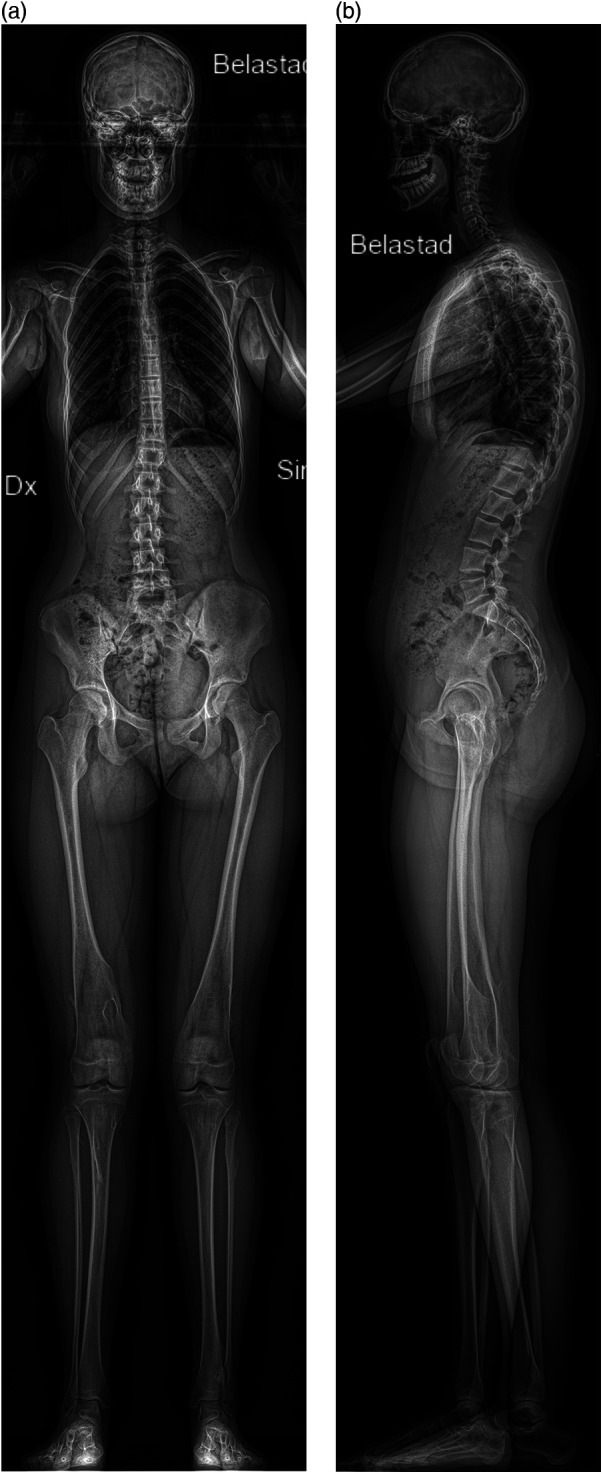
**(a,b)** Whole-body EOS images, frontal and laterally, of a 14-year-old girl. The forearms were not included within the examination field. For better visualization of the tibia and fibula, one foot was placed 5 cm dorsally on the lateral image.

Within a month after the appointment, all children underwent a whole-body examination (Figure 1a) using the EOS biplanar radiography system (EOS® imaging, ATEC Spine, Inc., Carlsbad, USA). The two examinations were made at different hospitals and could, for logistical purposes, not be done at the same appointment. The maximum time of one month between the examinations was set as a time frame where no significant growth of osteochondromas was likely to occur. The whole-body scanning included the whole lower limb from the hip joint to feet as well as the torso and skull, where the arms were positioned in 60 degrees of shoulder flexion and 90 degree of elbow flexion. Most children (8/10) were too large to position the entire forearms within the EOS apparatus and therefore, the osteochondromas in the forearms could not be reliably visualized.

The tube voltage was chosen by the radiographer among three different presets according to the patients’ weight and stature (small, medium, and large; 80–104 kV with 200–320 mA for anteroposterior projection and 100–120 kV with 200–320 mA for lateral projection), resulting in an average dose-area-product of 0.4 ± 0.1 mSv. The EOS system acquired simultaneously antero-posterior and lateral linear radiographs to map weight-bearing malalignment of the hip, knee and ankle joint and identify bone lesions visible in the same scan. These biplanar images were obtained in a standardized manner with one foot positioned 5 cm dorsally to better visualize the lower leg in the lateral projection (Figure 1b). After the EOS examination was concluded, all raw data images were sent to the vendor-specific workstation sterEOS (EOS® imaging, ATEC Spine, Inc., Carlsbad, USA) for reconstruction of EOS images.

Aside from the EOS examination, no additional radiographic imaging was conducted beyond what is included in nationally standardized protocols.

To evaluate the experience of the child's subjective experience of the examination, a set of questions referring to comfort, anxiety, and overall procedural tolerance directly were answered by all children directly after the examination. This questionnaire was developed for the purpose of this study, in a language adapted to children, as no suitable, previously validated Patient Reported Experience Measure (PREM) questionnaire was found (see Supplementary file).

### Radiographic interpretation

All radiographic interpretation was made by a senior radiologist with more than eight years’ experience of pediatric musculoskeletal radiology but without previous experience of the EOS method. First, the full leg weight-bearing frontal radiographs were assessed for presence of any osteochondroma >1 cm (as measured on the radiograph). Then, all osteochondroma on the EOS images in the pelvic area or in the lower extremities were noted both on the frontal and lateral views.

To ensure accurate comparison of osteochondroma size and counts between EOS biplanar imaging and conventional radiographs, a magnification correction was applied to account for differences in image scale between the two modalities. This correction was necessary because EOS imaging, due to its slot-scanning technology, has a lower inherent magnification factor compared to conventional digital radiographs.

First, the distance between the medial and lateral femoral condyles was measured on the frontal plane images of both modalities for each patient. This anatomical reference was chosen because it is reliably visualized on both conventional radiographs and EOS images and is not affected by minor postural differences.

Then, the magnification factor (MF) for EOS images was calculated for each patient as:

$$MF=\frac{Femoral\;condyle\;distance\;on\;conventional\;radiograph}{Femoral\;condyle\;distance\;on\;EOS\;image}$$

This provided a patient-specific correction factor reflecting the relative scale difference between the two imaging modalities. The measured size of each osteochondroma on EOS images was multiplied by the corresponding magnification factor to obtain a corrected size comparable to that measured on conventional radiographs:

Correctedosteochondromasize=MeasuredEOSsize×MF

Using this correction, osteochondromas that were ≥1 cm on conventional radiographs corresponded to ≥0.7 cm on uncorrected EOS images, ensuring that lesion identification thresholds were consistent between modalities.

**Figure 2:**
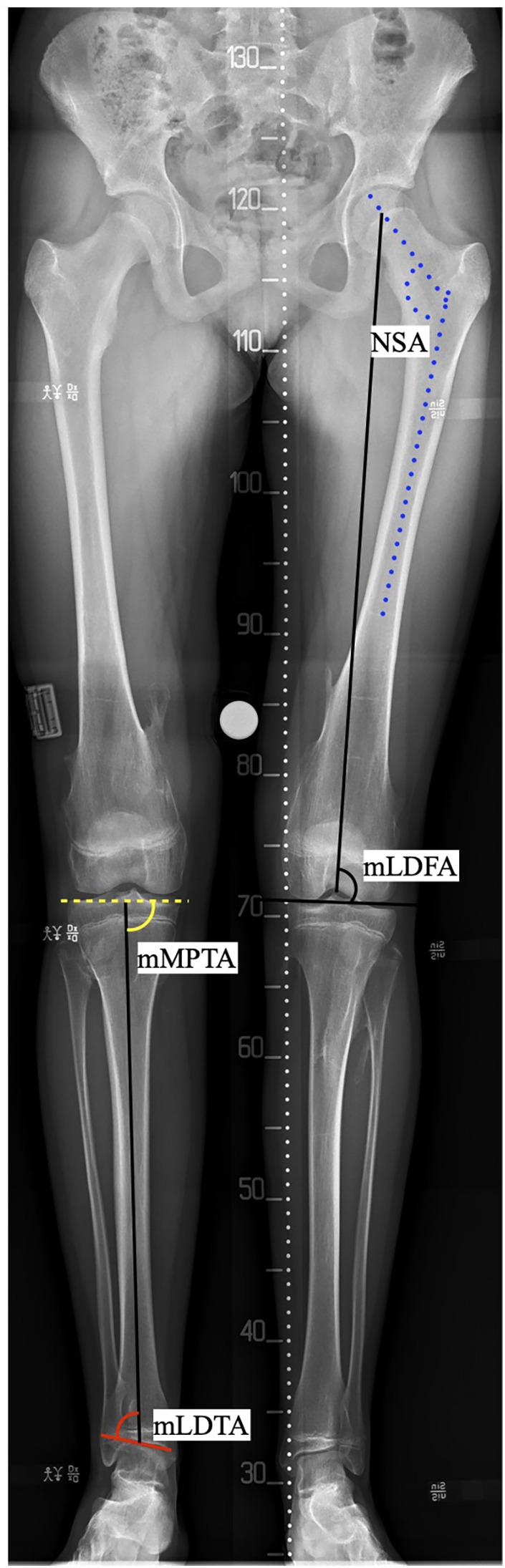
Measurements of frontal alignment. NSA, neck shaft angle; mLDFA, mechanical lateral distal femoral angle; mMPTA, mechanical medial proximal tibial angle; mLDTA, mechanical lateral distal tibial angle.

Further, the alignment of the hip, knee and ankle joint were assessed first on both the plain radiographs and the EOS images using standardized measurements and angles (Figure 2). All images were analyzed at a second occasion after 14 days by the same observer, blinded to earlier results. Although not part of the primary objective of the present study, osteochondromas that were identified in the torso or upper extremity (<1 cm) were noted.

### Statistics

Descriptive statistics were presented as absolute and relative frequencies of identified number of osteochondroma findings. Analysis comparing plain radiographs to EOS only included osteochondromas and measurements of the pelvis and lower extremities. Because of the small sample size, a Wilcoxon signed-rank test was employed to compare the number of osteochondromas identified by the two modalities.Osteochondromas noted in the torso or upper extremities were reported descriptively but were not included in the quantitative comparison, as no standardized radiographic reference was available for these regions. Bland-Altman plot analysis was performed to detect possible systematic biases and graphically visualize agreements between modalities.

The intra-observer agreement between repeated measurements was assessed by the coefficient of variation and displayed by correlation plots.

## Results

Ten, out of the 14 eligible children, as well as their parents, consented to participate while four children declined. The study group (median age 14.5; range 11–20) consisted of six males (14.5;11–20) and four females (13;10–14).

**Figure 3:**
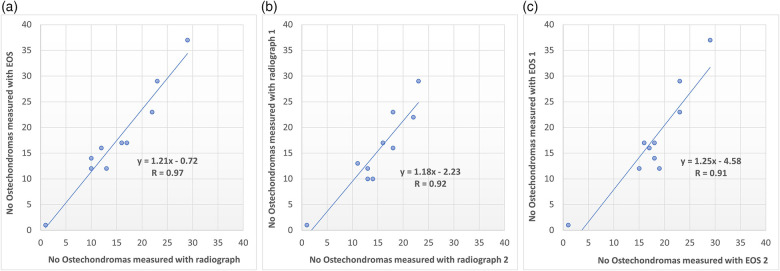
**(a)** Agreement between conventional radiographs and EOS of detected number of osteochondromas, as well as agreement in repeated counting of osteochondromas in **(b)** conventional radiographs and **(c)** EOS images.

In the lower extremities more osteochondromas were systematically identified with EOS compared to conventional radiography in the first measurement 16.3% as well as in the second 20.1%, *p* = 0.0242 and 0.0172 respectively (Table 1 and Figure 3). Figure 3 further shows that the number of identified osteochondromas was higher in the first measurements than in the last for both modalities.

**Table 1:** Number of identified osteochondromas during the first (M1) and second (M2) measurement for EOS and conventional radiography.

Study-ID	EOS		Conventional radiography		Difference *n* (%)		*p*-value	
	M1	M2	M1	M2	M1 EOS vs. M1 Radiograph	M2 EOS vs. M2 Radiograph	M1 EOS vs. M1 Radiograph	M2 EOS vs. M2 Radiograph
1	1	1	1	1	25 (16.3)	30 (20.1)	*p* = 0.0242	0.0172
2	14	18	10	13				
3	12	15	13	11				
4	17	18	17	16				
5	12	19	10	14				
6	37	29	29	23				
7	16	17	12	13				
8	23	23	22	22				
9	17	16	16	18				
10	29	23	23	18				
Total (mean;SD)	178 (17,8;9,94)	179 (17,9;7,26)	153 (15,3; 7,97)	149 (14,9;6,26)				

Wilcoxon signed-rank test was used for the *p*-values.

The EOS images of the torso and upper extremities provided additional visualizations of regions like the proximal humerus, costae and scapula. Out of the ten included patients, six had a total of 18 osteochondromas > 1 cm in these regions while no lesions were observed in the remaining four. Fourteen of the findings were observed in the humerus and scapula, while the remaining were identified in the ribs or forearm. Since no standardized upper body radiographs are part of the national follow-up protocols, these findings were not compared to plain radiographs. All of these identified osteochondromas had not been noted before.

**Figure 4:**
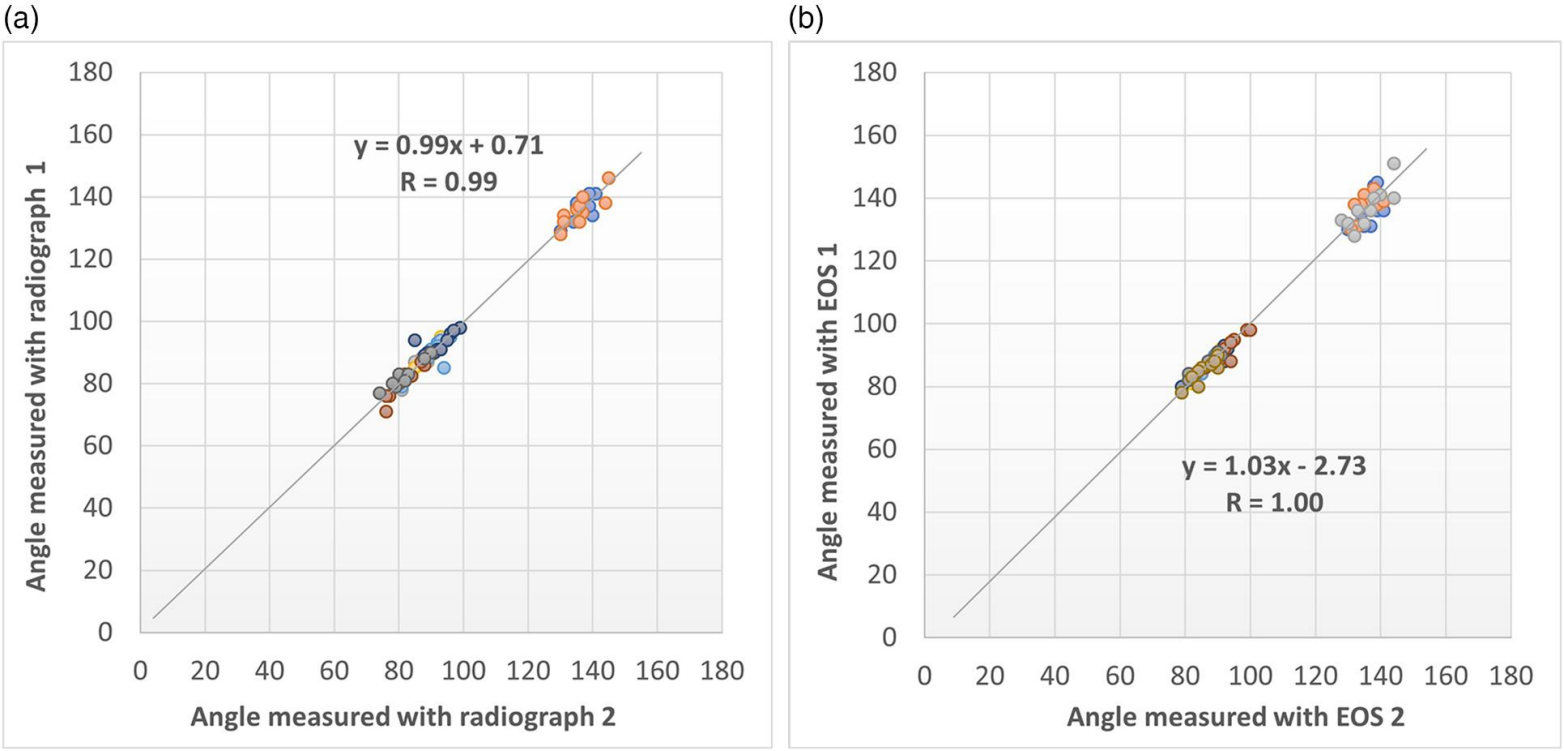
Agreement in repeated angle measurements performed on **(a)** conventional radiographical and **(b)** EOS technique.

**Figure 5:**
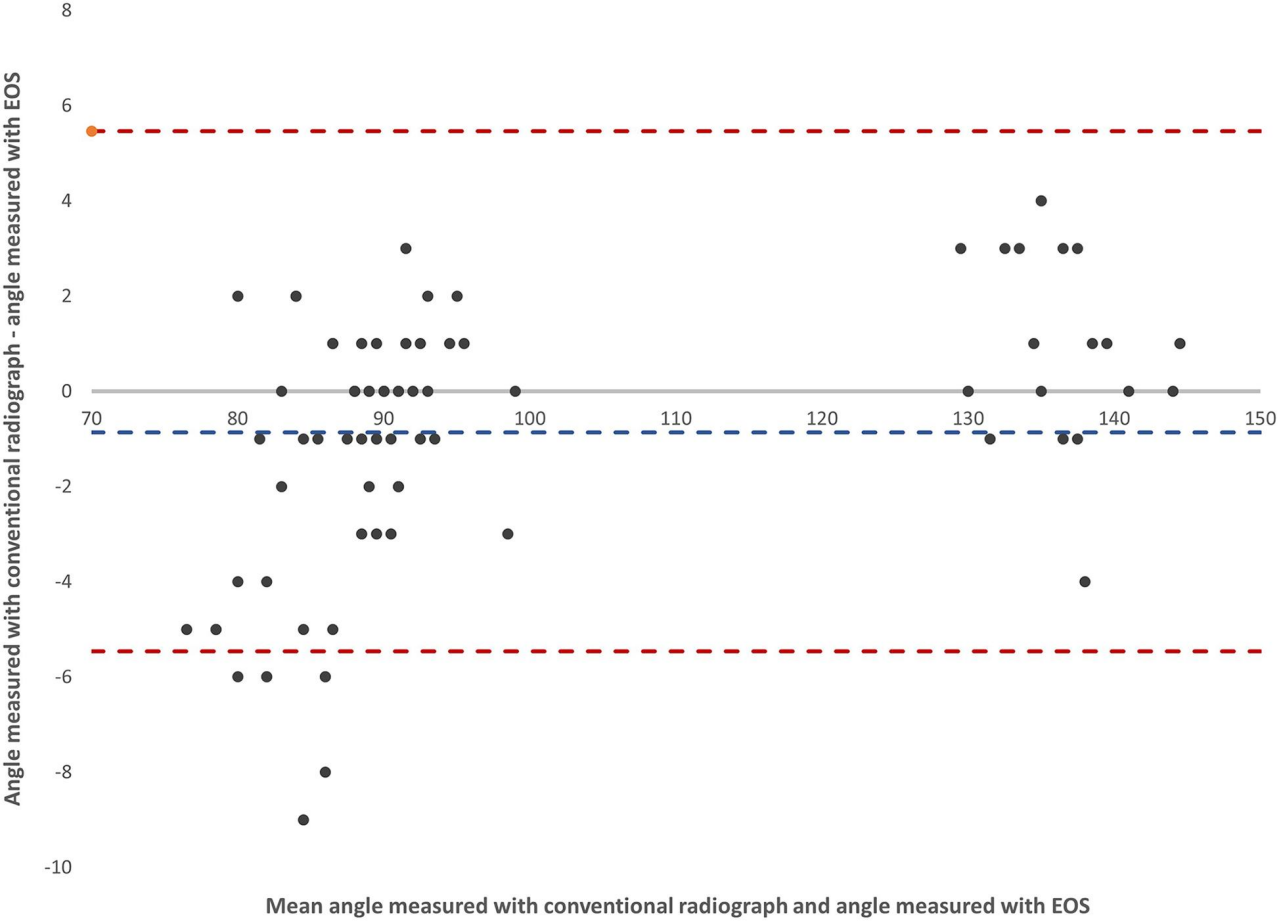
Bland–Altman plot for comparison of agreement between conventional radiographic and EOS angle measurements.

The Bland-Altman plot for the angle measurements displayed only a small systematic bias with narrow limits of agreement (LOA) between the conventional radiograph and EOS technique (Figure 4). The mean difference between the radiographs and EOS measurements was −0.86 degrees (95% LOA: −6.36–4.63 degrees), where LOA outliers included two cases. Also, the agreement between repeated angle measurements was found to be high (Figure 5). As shown by Figure 5 a very strong relationship was found between the repeated measurements for both modalities. Additionally, the coefficient of variation demonstrated equally good intra-observer agreement for both modalities (CV_radiographs_ = 2%; CV_EOS_ = 1%).

The PREM questionnaire revealed that the examination was reported as quick, easy and painless for almost all children (Table 2). One child complained that it was uncomfortable to stand still during the examination and 4/10 found it challenging not to move. Among the eight children who had previously, at some time, experienced a CT scan, seven preferred the EOS examination although no statistical comparison was made.

**Table 2:** Questions and obtained answers of the used PREM questionnaire (translated by the authors).

Question	Reply
Do you know what the machine is called?	Yes: 7, No: 3
Did what happen surprise you?	Yes: 2, No: 8
Were there too many instructions?	Yes: 0, No: 10
Tell us what it felt like? You may choose as many options as you like!	There were no problems: 8 It was difficult to stand still: 4 It hurt or was uncomfortable: 1
Did you have to wait for too long?	It was a quick examination: 8 It was ok: 2 The examination took too much time: 0
Have you done any similar (like CT) examinations before?	Yes: 8 No: 2
If you have done a CT-scan or normal x-ray examination before, what did you prefer?	The other examination (CT or normal x-ray): 1/8 The examination we did today (EOS): 7/8
Tell us what you liked or did not like with the examination today (EOS)	“Everything went well, it was fun to be raised and lowered” “It all went quickly and very smoothly” “All was fine, smooth” “Lots of noise, no need to change position” “Uncomfortable position that was difficult to hold” “I liked that it was quick” “It was quick and easy, nothing I disliked” “It was a bit tricky with the position, but it was quick and easy, better than normal radiographs”
How did it feel before the examination?	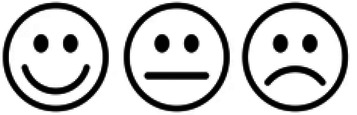 7 3 0
How does it feel after the examination?	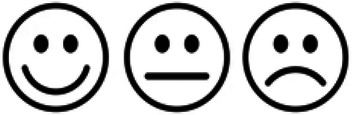 10 0 0

## Discussion

The results suggest that EOS biplanar imaging is a promising method to screen children with HMO. EOS could visualize more ostechondromas than conventional weight-bearing radiographs in the lower extremities, which is to be expected since it also provides a lateral view.

Even though EOS is not the optimal choice for visualization of osteochondromas in the spine, ribs or scapulae, our findings showed promise that EOS could visualize a number of ostechondromas in the torso and humerus that had not been noted previously on the clinical radiograph examination. Further, the malalignment in the frontal plane of the lower extremities was found to be reliably and reproducibly represented by EOS. The comparable diagnostic performance combined with the lower radiation exposure, makes it a favorable option for screening children with HMO.

It should be noted that smaller ostechondromas cannot be identified neither with conventional radiographs nor with EOS. However, the intended purpose with the screening was to identify osteochondromas large enough to cause mass induced symptoms or tethering and 1 cm was chosen as a cut-off for this purpose. EOS, with the higher achievable resolution in the biplanar image planes, seems to have higher feasibility to identify such ostechondromas (17). All osteochondromas found during the screening were not clinically relevant but such cases provide the opportunity to follow growth morphological changes of the detected osteochondromas over time, possibly making decisions for timely surgery easier.

No previous studies have used EOS biplanar imaging to screen children with HMO. The reliability and reproducibility of lower limb alignment measurements in this paper is in line with previous research on adults. Guggenberger et al. compared CT to upright full-length radiography and EOS biplanar imaging and found the results to be interchangeable as well as high interobserver agreement for all modalities (18). EOS has in another study been shown to enable more accurate leg length measurements than conventional radiographs (19). Regarding pelvic anatomy, Bittersohl et al. found that EOS proved reliable for the assessment of gross pelvic and acetabular morphology; a finding relevant to the present indication since acetabular morphology may be affected by coxa valga secondary to HMO. While the present study did not examine lower limb rotational alignment, EOS has previously been proven to be comparable to CT for this indication (20) and, as such, may be useful also for HMO screening.

Internationally, the protocols for HMO screening vary greatly both in children and adults, with limited scientific support. Also, most previous papers relate to the screening of adults and as stated above, such screening has different indications. In our country, a national consensus regarding the radiographic follow-up of children with HMO state that these children should undergo a conventional full-length standing anteroposterior radiograph every second year and a conventional radiograph of the hips and pelvis (including lateral imaging of the hips) every fourth year. Additionally, any new osteochondromas that cause local symptoms should be subjected to radiographs. CT is recommended to be used only for preoperative planning of complex osteochondromas and, using an ultra-low-dose protocol, to initially verify the diagnosis. These national screening guidelines were created to decrease the child's exposure to radiation considering the high total radiation exposure during childhood for children with HMO and the associated increased risk for malignancy.

EOS may primarily replace the full-length standing anteroposterior radiographs as well as the radiographs of the pelvis and hip. This may further decrease the total radiation exposure minimizing the need for additional targeting radiographs of palpable osteochondromas not visible on frontal radiographs. This is in line with a recent report that suggests replacing standard radiographs with EOS imaging for scoliosis follow-ups in children.

**Figure 6:**
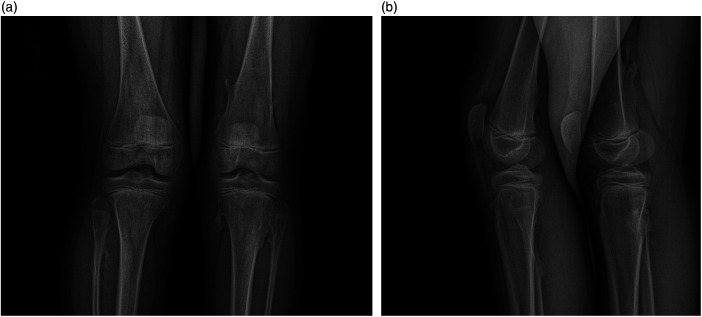
**(a,b)** A frontal and lateral EOS image of the knee in a 10-year-old boy. Multiple osteochondromas are clearly visible.

The reproducibility findings showed that the radiologist consistently identified more osteochondromas in the first interpretation than in the second. This was true for both modalities. The difference between the repeated interpretations most probably reflected the learning curve of the radiologist. According to the radiologist, the spikes of an osteochondroma could, at first impression, be interpreted as separate even though they branch out from the same base (Figure 6).

It is well known that EOS images have relative lower magnification compared to the plain radiographs. This discrepancy was easily adjusted as described above. Also, the magnification of the objects in the sagittal EOS images differs somewhat between the two legs. This small discrepancy was not adjusted, as it was considered to be of no significance for the purposes of this study; however, it may be relevant when estimating the true size of osteochondromas using a reference bead.

While the ability to create 3D models from biplanar EOS images is less useful for HMO screening, the feature is a great advantage in the diagnoses of e.g., scoliosis or certain extremity malalignments and requires markedly less exposure compared to a CT scan (21). The 3D images of the lower extremities are produced by marking certain pre-set anatomical landmarks as points of reference to create a computer produced image based on a standardized bone. The technique could, of course, be used to better visualize sagittal and rotational malalignments caused by the osteochondromas, but normally malalignments in the frontal plane is the main challenge in this diagnosis.

Overall, the children reported the examination to be easy, comfortable and as convenient as a standing radiographical investigation, and most preferred EOS to a supine CT-scan. This further strengthens EOS as a viable screening tool for children with HMO.

### Limitations

This explorative study aimed to display the feasibility of EOS for HMO screening and as such was limited in sample size. Future large cohort studies are warranted to fully establish the diagnostic certainty of the EOS method.

Also, the study was limited to compare EOS with plain radiographs, since it is the established method for HMO screening in our clinic. In similar to EOS, such images are acquired during weight-bearing without exposing the children to unacceptable levels of radiation as in CT scan.

In addition, the agreement analysis was limited to only one examiner and although the intra-observer reliability was excellent, no inter-observer reliability was established.

The >1 cm size cutoff was author-defined and therefore carries some subjectivity. The purpose of the PREM questions was exploratory for a novel examination modality rather than to reliably compare patient-reported experience between modalities.

## Conclusions

EOS biplanar imaging showed good feasibility and seemed to be a reliable and reproducible technique to screen children with HMO for identification of osteochondromas of the lower extremities and in the same investigation provide information on deformities. The examinations were quick and well tolerated by the children with a low exposure to radiation.

## Data Availability

The raw data supporting the conclusions of this article will be made available by the authors, without undue reservation.
