# Supplementary material for: Biplanar EOS screening in children with hereditary multiple osteochondromas: a feasible screening method?
Source: Front Pediatr. 2026 Jan 12;13:1625991. doi: 10.3389/fped.2025.1625991 (PMC12833365; doi:10.3389/fped.2025.1625991)
Supplement: Supplementary file 1 [file Table1.docx]

Hej!
Du har just blivit undersökt med vår nya röntgenkamera, som kallas EOS.

Vi vill gärna veta vad du tycker om den. Därför vill vi att du svarar på några frågor:

|  | Fråga | Sätt ett kryss för det svar du väljer |
| --- | --- | --- |
| 1 | Fick du reda på vad maskinen heter? | Ja  Nej |
| 2 | Blev du förvånad över vad du fick göra? | Ja  Nej |
| 3 | Blev det för många instruktioner? | Ja  Nej |
| 4 | Berätta något om hur du kände?  Du får välja så många svar du vill! | Det var svårt att stå stilla hela tiden  Det gjorde ont  Jag var rädd  Jag tyckte det var obehagligt/läskigt  Det var bullrigt  Det var inga problem att bli undersökt med EOS  Annat, skriv här: |
| 5 | Blev det mycket väntan utan något att göra? | Det tog alldeles för lång tid  Det gick snabbt  Det var väl ok |
| 6 | Har du gjort det här förut? | Ja  Nej |
|  | Om du har gjort det här tidigare vilken gillar du bäst? | Maskinen innan (Skiktröntgen eller ”vanlig” röntgen)  Maskinen idag (EOS) |
| 7 | Berätta för oss vad du gillade och ogillade med maskinen idag (EOS) |  |
| 8 | Hur kändes det innan? | 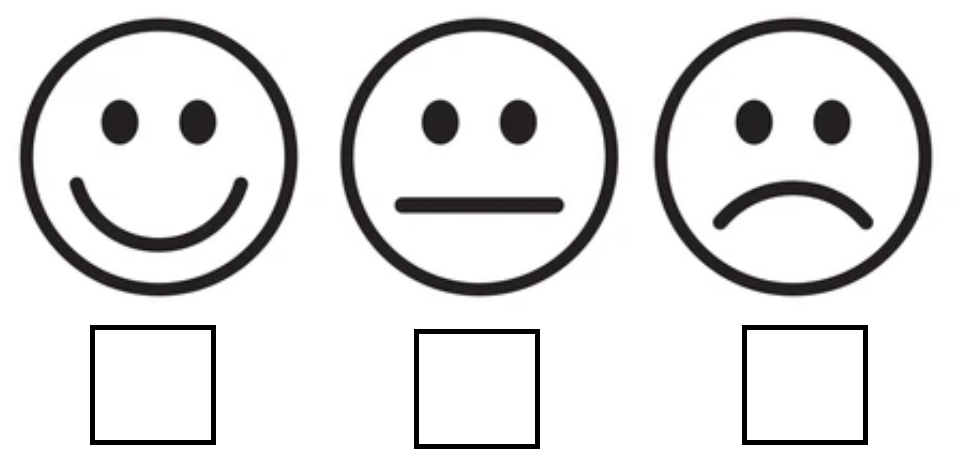 |
| 9 | Hur kändes det efter? | 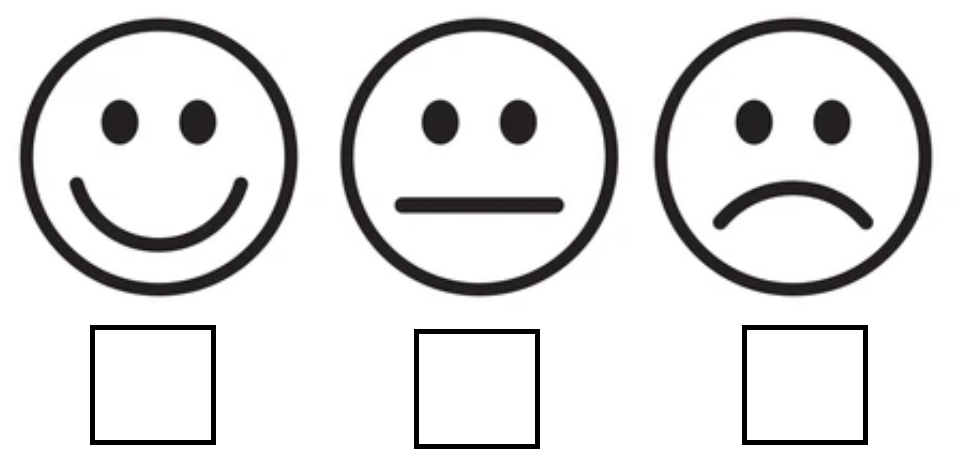 |

**Tack för att du svarat!**

Lämna tillbaka blanketten ifylld.

Med vänlig hälsning

Henrik Hedelin, studieansvarig
